# Specimen Identification Through Multilocus Species Tree Constructed From Single‐Copy Orthologs (SCOs): A Case Study in *Cymbidium* Subgenus *Jensoa*

**DOI:** 10.1002/ece3.71323

**Published:** 2025-04-24

**Authors:** Zheng‐Shan He, Ji‐Xiong Yang, Jia‐Lin Huang, De‐Zhu Li, Jun‐Bo Yang

**Affiliations:** ^1^ Germplasm Bank of Wild Species & Yunnan Key Laboratory of Crop Wild Relatives Omics Kunming Institute of Botany, Chinese Academy of Sciences Kunming Yunnan China; ^2^ Yuxi Normal University Yuxi Yunnan China

**Keywords:** closely related species, *Cymbidium* subgenus *Jensoa*, pipeline, single‐copy orthologs (SCOs), specimen identification

## Abstract

Standard barcodes and ultra‐barcode encounter significant challenges when delimiting and discriminating closely related species characterized by deep coalescence, hybrid speciation, gene flow, or low sequence variation. Single‐copy orthologs (SCOs) have been widely recognized as standardized nuclear markers in metazoan DNA taxonomy, yet their application in plant taxonomy remains unexplored. This study evaluates the efficacy of SCOs for identifying recently diverged species within the *Cymbidium* subgenus *Jensoa*, where ultra‐barcodes have previously shown limited resolution. Remarkably, over 90% of the 9094 targeted reference SCOs, inferred from three *Cymbidium* genomes, were successfully retrieved for all 11 representative species in subg. *Jensoa* using ALiBaSeq at a minimal 5× depth from whole genome shotgun sequences. The species tree, reconstructed from multiple refined SCO matrices under the coalescent model, effectively distinguished all species and identified mislabeled or misidentified specimens. The comprehensive and refined SCO matrices produced by our pipeline not only enhance phylogenetic analysis but also improve the precision of species diagnosis. Additionally, biparentally inherited SCOs, serving as multi‐locus markers, not only augment the effectiveness of DNA barcoding but also support a transition to multi‐locus, species‐tree‐based specimen assignment strategies.

## Graphical abstract

Standard DNA barcodes often fail to distinguish closely related plant species due to deep coalescence, hybridization, and low‐sequence variation. In this study, we demonstrate that single‐copy orthologs (SCOs), widely used in metazoan taxonomy, can serve as effective multilocus markers for plant species identification. Using *Cymbidium* subgenus *Jensoa* as a case study, we show that SCO‐based species trees significantly improve species resolution and specimen identification, offering a powerful alternative to traditional barcode methods.

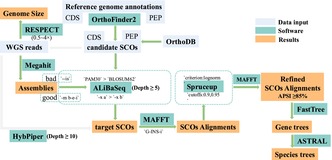

## 1 Introduction

Species recognition is crucial for both scientific research and societal applications (Karbstein et al. 2024). Proposed by Hebert 21 years ago (Hebert et al. 2003), DNA barcoding has proven to be a vital tool for the identification and discovery of plant species through the genetic variations in DNA sequences (Hollingsworth et al. 2016). Consensus has been reached on four readily amplified gene regions—*rbc*L, *mat*K, *trn*H‐*psb*A, and ITS (internal transcribed spacers)—as the standard plant DNA barcodes (Hollingsworth et al. 2009; Kress et al. 2005; Li et al. 2011). However, standard barcodes often fail in many evolutionarily young species due to insufficient sequence divergence (Li, Yang, et al. 2015; Spooner 2009; van Velzen et al. 2012). Ultrabarcoding (UBC), utilizing whole chloroplast genome (Kane and Cronk 2008) or ribosomal DNA (rDNA) repeat units (Kane et al. 2012) as extended barcodes, has circumvented the intrinsic limitations of single‐ or multi‐locus DNA barcodes by providing a plethora of variable characters (Coissac et al. 2016). This technique, leveraging low‐coverage shotgun sequencing for the assembly of plastomes and rDNA clusters, renders the use of universal primers and loci preference negligible (Kress et al. 2005; Straub et al. 2012). Ultrabarcoding has thus emerged as a more discriminating and efficient method for resolving complex plant taxa (Ji et al. 2019; Jiang et al. 2024; Kane et al. 2012; Parks et al. 2009; Ślipiko et al. 2020; Yang et al. 2013; Zeng et al. 2018). Nevertheless, challenges persist in distinguishing species affected by introgression, hybridization, incomplete lineage sorting (ILS), or recent divergence through plastomes and rDNA repeats alone (Ruhsam et al. 2015; Weitemier et al. 2014). Polyphyly or paraphyly at the species level remains prevalent among closely related groups, particularly those that have diverged recently (Dupuis et al. 2012; Liu, Ma, Ci, et al. 2021; van Velzen et al. 2012; Yu et al. 2022).

DNA barcoding typically depends on the concept of a “barcode gap,” which refers to the distinction between the minimal interspecies genetic distance and the maximal intraspecies distance (Puillandre et al. 2012). However, the existence of a barcode gap is influenced not only by genetic divergence between species but also by the absence of incomplete lineage sorting (ILS) events and gene flow (Mallo and Posada 2016). Furthermore, there is no universally applicable threshold for all taxa; these thresholds are subjective and challenging to determine a priori (Brown et al. 2012; Moritz and Cicero 2004). A simulation study demonstrated that methods relying on a single‐threshold barcode performed poorly, with coalescence methods significantly outperforming them (Yang and Rannala 2017). It is suggested that the organellar genome be treated as a single “locus” in multi‐locus species tree analyses due to its genealogical homogeneity (Flouri et al. 2018). A “next‐gen” barcoding framework has been proposed, integrating multilocus data sets with multispecies coalescent‐based methods for accurate specimen identification (Dowton et al. 2014). However, a case study failed to adequately demonstrate the shortcomings of standard DNA barcoding due to the arbitrary selection of a 4% K2P divergence threshold (Collins and Cruickshank 2014). Two example data sets where the COI barcoding approach was inadequate showed that closely related species could be accurately identified using a multilocus barcoding method after analyzing hundreds of nuclear gene markers (Liu et al. 2017).

Nuclear genes, notable for their predominantly biparental inheritance compared to organelle genes, can significantly enhance the accuracy and robustness of DNA barcoding (David et al. 2021; Huang et al. 2022; Small et al. 2004; Wang et al. 2019; Zimmer and Wen 2012). However, ITS and rDNA can fail to accurately represent both parent genomes in hybrids and allopolyploids due to the lack of intragenomic uniformity and their complex evolutionary paths (Álvarez and Wendel 2003; Bailey et al. 2003). Furthermore, ultra‐conserved elements (UCEs) and restriction site‐associated DNA (RAD) sequencing face challenges due to insufficient intraspecific variation or sequences in nonhomologous flanking regions (Eberle et al. 2020). Advances in sequencing technologies have disrupted the trade‐off between cost and accuracy of barcoding results. Since the publication of the first plant reference genome of *Arabidopsis thaliana* in 2000, over 3000 genome assemblies from numerous plant species have been sequenced (Xie, Gong, et al. 2024). Additionally, the One Thousand Plant Transcriptomes Initiative (OneKP) has published more than 1000 transcriptomes, significantly advancing our understanding of plant genomics (Leebens‐Mack et al. 2019). Among the tools emerging from these efforts, the Angiosperm353 nuclear probe set (Johnson et al. 2018; McLay et al. 2021), derived from a wide array of orthologous loci identified by OneKP, has been extensively utilized. These probes enable the comprehensive capture of nuclear markers across a broad range of plant taxa, facilitating detailed phylogenetic studies and species identification (Zuntini et al. 2024). Techniques such as whole transcriptome sequencing, targeted enrichment, and whole‐genome sequencing have now become affordable, enabling the sampling of hundreds of single‐copy target loci from the nuclear genome (Lemmon et al. 2012; Weitemier et al. 2014; Wen et al. 2013; Xi et al. 2013).

Single‐copy orthologs (SCOs) are protein‐coding genes that are strongly selected to exist in only one copy per genome, enhancing the reliability of homology assessments and making them exceptionally suitable and universal markers (Waterhouse et al. 2011). The quantity of SCOs tends to increase with the relatedness of the species studied, resulting in a higher number of inferred SCOs at lower taxonomic levels than at higher lineages (Emms and Kelly 2019; Smith and Hahn 2021). Putative SCOs can be recovered in two ways: (a) identifying and assembling corresponding reads from reference SCOs or (b) assembling the entire genome and then extracting each putative SCO by querying them against the whole assembly (Knyshov et al. 2021). SCOs have consistently enhanced and standardized species delimitation and discrimination within Metazoa (Dietz et al. 2023; Joshi et al. 2022). Although single‐copy orthologs (SCOs) have been widely used as molecular markers in plant phylogenetic studies for several years (Hu et al. 2023; Huang et al. 2022; Johnson et al. 2018; Liu, Ma, Ren, et al. 2021; Liu et al. 2022; Zhang, Hu, et al. 2023; Zhang et al. 2012), their application in specimen identification remains unexplored.

Subgenus *Jensoa* (Raf.) Seth & Cribb (Orchidaceae; Epidendroideae; Cymbidieae; Cymbidiinae; *Cymbidium*) comprising approximately 20 species, predominantly consists of terrestrial plants growing in tropical and subtropical Asia (Liu et al. 2006; Zhang et al. 2021). The well‐known Asian Cymbidiums, which have been cultivated in China for over 2000 years, originate from this subgenus and include thousands of artificial hybrids (Du Puy et al. 2007; Hew 2001). Subgenus *Jensoa* diverged less than 4 million years ago (Chen et al. 2024; Zhang, Hu, et al. 2023), and its species exhibit minimal morphological variation prior to flowering. Given that hybridization is as prevalent as poaching within *Jensoa*, accurate identification of this subgenus is crucial for breeding and trade (Liu et al. 2006). Previous attempts have failed to accurately identify this group using standard DNA barcodes and ultrabarcodes, such as plastomes and unassembled reads (Zhang, Huang, et al. 2023). As a demonstration of how SCOs can be utilized, we will explore the effectiveness of SCOs in distinguishing recently diverged species of the *Cymbidium* subgenus *Jensoa* (Orchidaceae), which frequently undergo hybridization. Lineage‐specific reference SCOs were initially inferred from three annotated whole genomes of species within *Cymbidium*. Subsequently, putative SCOs were recovered from deep genome skimming data of 11 *Jensoa* species with multiple samples. This study aims to address three key questions: (i) Is it feasible to recover the vast majority of SCOs from genomic sequencing data with less than 10× depth? (ii) How can we obtain reliable SCO matrices and subsequent species trees using a convenient pipeline? and (iii) What are the feasibility and advantages of SCOs in plant specimen identification compared to existing DNA barcoding?

## 2 Materials and Methods

### 2.1 Plant Material and Data Collection

According to our recent study (Zhang, Huang, et al. 2023), 11 polyphyletic species of *Cymbidium* subgenus *Jensoa* were selected for analysis. Initially, four individual representatives of each species were sequenced, yielding approximately 100 Gb of genomic data. Of these, 33 of the 44 vouchers were identical to those used in our previous research (Zhang, Huang, et al. 2023). Additionally, *C. mannii* from subgenus *Cymbidium* (Fan et al. 2023), *C. tracyanum* from subgenus *Cyperorchis*, both part of our comparative genomics project on *Cymbidium*, were included as the closely related outgroups. As distantly related outgroups, three species from the same tribe Cymbidieae were selected: *Grammatophyllum scriptum* and *Thecopus maingayi* from subtribe Cymbidiinae, and *Acriopsis javanica* from subtribe Acriopsidinae. To further assess intraspecific genetic variation in *C. ensifolium*, three additional collections (H3204, ZL442, and ZL443) and another published collection (Voucher RL0671, accession SRR7121924) (Liu et al. 2019) were added to the study (Table 1). The methods for DNA extraction and genomic sequencing followed previously described protocols (Zhang, Huang, et al. 2023), with the exception that genome sequencing was conducted using the DNBSEQ‐T7 platform. The raw data were filtered using Fastp v0.22.0 with default parameters (Chen et al. 2018).

**TABLE 1:** Species information for all materials utilized in this study.

Species	Voucher	Locality	Clean data (Gbp)	Genome size (Gb)	Sequencing depth
*Cymbidium tortisepalum*	18HT2037	Lijiang, Yunnan, China	115.80	3.64	31.81
	ZL55	KBG, Yunnan, China	118.75		32.62
	ZL56	Baoshan, Yunnan, China^b^	113.30		31.13
	ZL70	Dali, Yunnan, China	114.29		31.40
*C. goeringii*	15043	Enshi, Hubei, China	132.81	4.88	27.21
	16264	Chongqing, China	102.04		20.91
	16266	Chongqing, China^b^	110.48		22.64
	16280	Chongqing, China^b^	115.07		23.58
*C. serratum*	H4001	Baise, Guangxi, China	100.78	3.70	27.22
	H4002	Baise, Guangxi, China^b^	104.15		28.13
	H4003	Baise, Guangxi, China^b^	125.60		33.92
	ZL453	Qianxinan, Guizhou, China^b^	109.45		29.56
*C. omeiense*	15002	Zhangjiajie, Hunan, China^b^	120.23	3.82^d^	31.46
	15009	Zhangjiajie, Hunan, China^b^	102.52		26.83
	15032	Enshi, Hubei, China^b^	130.21		34.07
	15034	Enshi, Hubei, China^b^	107.39		28.10
*C. kanran*	18HT1428	Honghe, Yunnan, China^b^	147.91	4.22	35.05
	18HT1873	Lijiang, Yunnan, China^b^	123.40		29.24
	H3602	Qianxinan, Guizhou, China^b^	95.08		22.53
	H3605	Qianxinan, Guizhou, China^b^	99.29		23.53
*C. faberi*	15019	Enshi, Hubei, China^b^	125.75	3.12	40.30
	15020	Enshi, Hubei, China^b^	149.05		47.77
	15030	Enshi, Hubei, China^b^	112.40		36.03
	ZL39	KBG, Yunnan, China	107.26		34.38
*C. sinense*	ZL3	Honghe, Yunnan, China^b^	102.69	4.62	22.22
	ZL4	Honghe, Yunnan, China^b^	110.76		23.96
	ZL444	Honghe, Yunnan, China^b^	114.16		24.70
	ZL445	Yunnan, China^b^	107.57		23.27
*C. qiubeiense*	19HT2776	Qianxinan, Guizhou, China^b^	160.75	6.19	25.97
	ZL13	Qianxinan, Guizhou, China^b^	170.09		27.48
	ZL14	Qianxinan, Guizhou, China^b^	135.91		21.96
	ZL457	Qianxinan, Guizhou, China	105.37		17.02
*C. cyperifolium* var. *szechuanicum*	ZL19	Qianxinan, Guizhou, China^b^	131.17	4.41	29.72
	ZL20	Qianxinan, Guizhou, China^b^	154.88		35.09
	ZL64	Qianxinan, Guizhou, China^b^	112.20		25.42
	ZL65	Baise, Guangxi, China^b^	146.16		33.12
*C. cyperifolium*	14,942	Hechi, Guangxi, China	90.55	4.09	22.14
	16,268	Chongqing, China^b^	102.68		25.10
	ZL21	Qianxinan, Guizhou, China^b^	103.54		25.32
	ZL22	KBG, Yunnan, China	105.78		25.86
*C. ensifolium*	13,553	Baise, Guangxi, China	107.19	3.18	33.76
	18HT2190	Linzhi, Xizang, China^b^	144.94		45.65
	H3201	Baise, Guangxi, China^b^	138.53		43.63
	H3202	Baise, Guangxi, China	112.05		35.29
	H3204^a^	KBG, Yunnan, China	30.45		9.59
	ZL442^a^	Wenshan, Yunnan, China^b^	31.95		10.06
	ZL443^a^	Nujiang, Yunnan, China^b^	31.46		9.91
	RL0671	Ruili, Yunnan, China^c^	73.50		23.15
Outgroup					
*C. mannii*	YYL1809	KBG, Yunnan, China	Chromosome‐level assembly	2.75	/
*C. tracyanum*	ZL1	KBG, Yunnan, China^b^	Chromosome‐level assembly	3.95	/
*Grammatophyllum scriptum*	Cymw4^a^	Taiwan, China^b^	56.09	/	/
*Acriopsis javanica*	Cymw6^a^	Thailand^b^	62.02	/	/
*Thecopus maingayi*	Cymw7^a^	Thailand^b^	31.30	/	/

^a^ Six additional individuals sequenced, each exceeding 25 Gb of genomic data; KBG, Kunming Botany Garden.

^b^ Vouchers identical to those used in our previous study (Zhang, Huang, et al. 2023).

^c^ Accessions derived from another study (Liu et al. 2019).

^d^ Genome size estimated according to RESPECT results, not by flow cytometry.

### 2.2 Genome Size Estimation

Genome size estimates for all samples were obtained using flow cytometry (FCM). Approximately 20 mg of fresh young leaf tissue was minced using a scalpel in a plastic Petri dish kept on the ice and containing 1 mL of ice‐cold nuclei isolation buffer (45 mM MgCl_2_·6H_2_O, 20 mM MOPS, 30 mM Trisodium citrate, 1% (W/V) PVP 40, 0.2% (V/V) Triton X‐100, and 10 mM Na_2_EDTA, pH 7.0). The homogenate was then filtered through a 42‐μm pore size nylon mesh into polystyrene sample tubes. The nuclear suspension was divided into three aliquots of 0.5 mL to which 50 μg/mL propidium iodide was added for staining, and RNase A (50 μg/mL) was then added to remove RNA contamination. Samples were incubated on ice in the dark for 1 h with occasional shaking to get well‐defined populations. All the samples were analyzed using a BD FACSCalibur Flow Cytometer, using the *Zea mays* B73 (2.3 Gb/1C, diploid) as the internal reference (Table S1).

For genomic analysis, 44 clean paired‐end genomic data sets were processed with JellyFish v2.3.0 (Marçais and Kingsford 2011) to compute a histogram of k‐mer frequencies for each sample using subcommand “jellyfish count‐C‐m21” and “jellyfish histo‐h 3000000.” GenomeScope v2.0 (Ranallo‐Benavidez et al. 2020) was then used to estimate the genome size of each sample using default parameters. Due to failures in some samples with GenomeScope2, original clean data from all individuals were subsampled to 0.5–4× coverage using seqtk v1.3‐r106 (Li 2012) and merging by BBMerge v39.01 (Bushnell et al. 2017). Subsequently, genome sizes for all individuals were estimated using RESPECT v1.3.0 (Sarmashghi et al. 2021) (Table S1).

### 2.3 Genome Assembling and SCOs Retrieval

To efficiently handle approximately 5 TB clean genomic data, ultrafast, memory‐efficient short‐read assemblers were selected for processing. Clean pair‐end reads were assembled using SOAPdenovo v2.04 (Luo et al. 2012) with the command “SOAPdenovo‐63mer all‐K 41” or MegaHit v1.2.9 (Li, Liu, et al. 2015) with default parameters. Protein annotations from three *Cymbidium* genomes (*C. tortisepalum*, *C. mannii* , and *C. tracyanum*) were analyzed using OrthoFinder v2.3.8 (Emms and Kelly 2019) to identify 9094 SCOs genes. These protein sequences were then employed as queries in TBLASTN searches against all short‐read assemblies and two chromosomal‐level assemblies. ALiBaSeq v1.2 (Knyshov et al. 2021) was used to extract these 9094 SCOs from the TBLASTN results, utilizing parameters “‐x a‐e 1e‐10–is–amalgamate‐hits–ac aa‐tdna.” To exclude introns identified by ALiBaSeq, the default scoring matrix of TBLASTN was modified to PAM30. Additionally, to evaluate the performance of ALiBaSeq at lower sequencing depths (below the 10× coverage recommended by Liu, Ma, Ren, et al. 2021), 25% subsampling was applied to the clean genomic data of all 44 individuals.

### 2.4 Chloroplast Genomes and nrDNA Assembling

Chloroplast genomes and nuclear ribosomal DNA (rDNA) clusters were de novo assembled using GetOrganelle v1.7.5 (Jin et al. 2020) and/or NOVOPlasty v4.3.1 (Dierckxsens et al. 2016). For reference, the plastome of *C. sinense* (accession: NC_021430) and the nrDNA of *C. macrorhizon* (accession: MK333261) were selected. The small single copies (SSCs) of all assembled plastomes were adjusted to the same direction as needed. When the nrDNA sequences of each individual were incomplete, they were manually concatenated based on the mapping results using Geneious R9 (Biomatters).

### 2.5 Alignment Filtering and Tree Building

**FIGURE 1:**
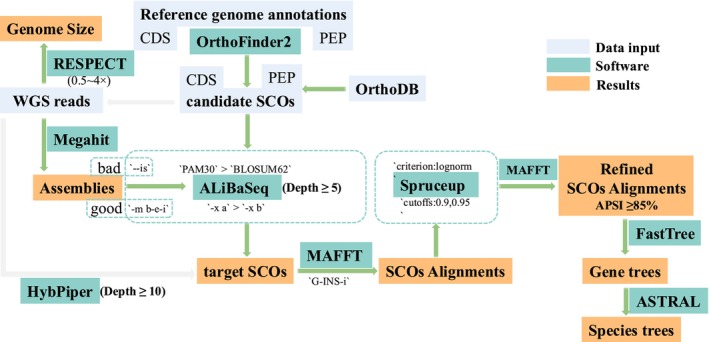
Graphical overview of study pipeline. Displaying the pipeline of this study using green arrows. Key software parameters are enclosed in single quotation marks. Gray arrows depict an alternative method for recovering putative SCOs, which has not been fully validated in this study. APSI, average pairwise sequence identity.

The single‐copy homologous genes matrix, recovered by ALiBaSeq, was aligned by MAFFT v7.508 with the “–globalpair” parameters (Katoh and Standley 2013). Average pairwise sequence identity (APSI) for each alignment, a metric measuring sequence homology, was computed utilizing ALISTAT v1.9g from the squid package (Eddy 2005). The ShannonEntropy of an alignment was calculated using the script ShannonEntropyScript.py, available at https://github.com/vinhbiochem/ShannonEntropy/. To mitigate the risk of including nonhomologous regions, Spruceup v2022.2.4 (Borowiec 2019) employed with a cutoff value of 0.95. Only alignments with no missing data and an APSI greater than 85% were selected for further analysis. Approximately maximum likelihood gene trees were constructed using FastTree v2.1.10 (Price et al. 2010) with parameters “‐gtr‐gamma–nt” based on these refined alignments. Species trees were subsequently inferred using ASTRAL v5.7.8, and normalized quartet scores were retrieved from logfiles (Mirarab et al. 2014) (Figure 1).

## 3 Results

### 3.1 Genome Sizes of Species in *Cymbidium* Subg. *Jensoa*

To accurately estimate the sequencing depth for each species, genome sizes were initially measured. Based on flow cytometry results, the average genome size for all 11 species of the subgenus *Jensoa* was 4.1 Gb, aligning with the mean value reported for *Cymbidium* in the Plant DNA C‐values database (Leitch et al. 2019). *Cymbidium qiubeiense* exhibited the largest genome at 6.19 Gb, whereas *C. faberi* and *C. ensifolium* had the smallest, approximately 3.1 Gb each (Table 1). Estimates provided by GenomeScope2 did not always align closely with flow cytometry results, potentially due to inadequate sequencing depth or incorrect k‐mer peaks selected by GenomeScope2. Calculations performed by RESPECT showed genome sizes approximately 1.19 times larger than those measured by flow cytometry. The coefficient of variation (CV) of genome sizes of each species estimated by GenomeScope2 was much higher than estimated by RESPECT (Table S1). Based on these genome size determinations, the sequencing depth for all 44 individuals ranged from 17.02× to 47.77× (average 29.46×), whereas the depth for the 25% subsampled data from the 44 individuals and three additionally included *C. ensifolium* specimens ranged from 4.26× to 11.94× (Table 1).

### 3.2 Putative SCOs Recovery

**FIGURE 2:**
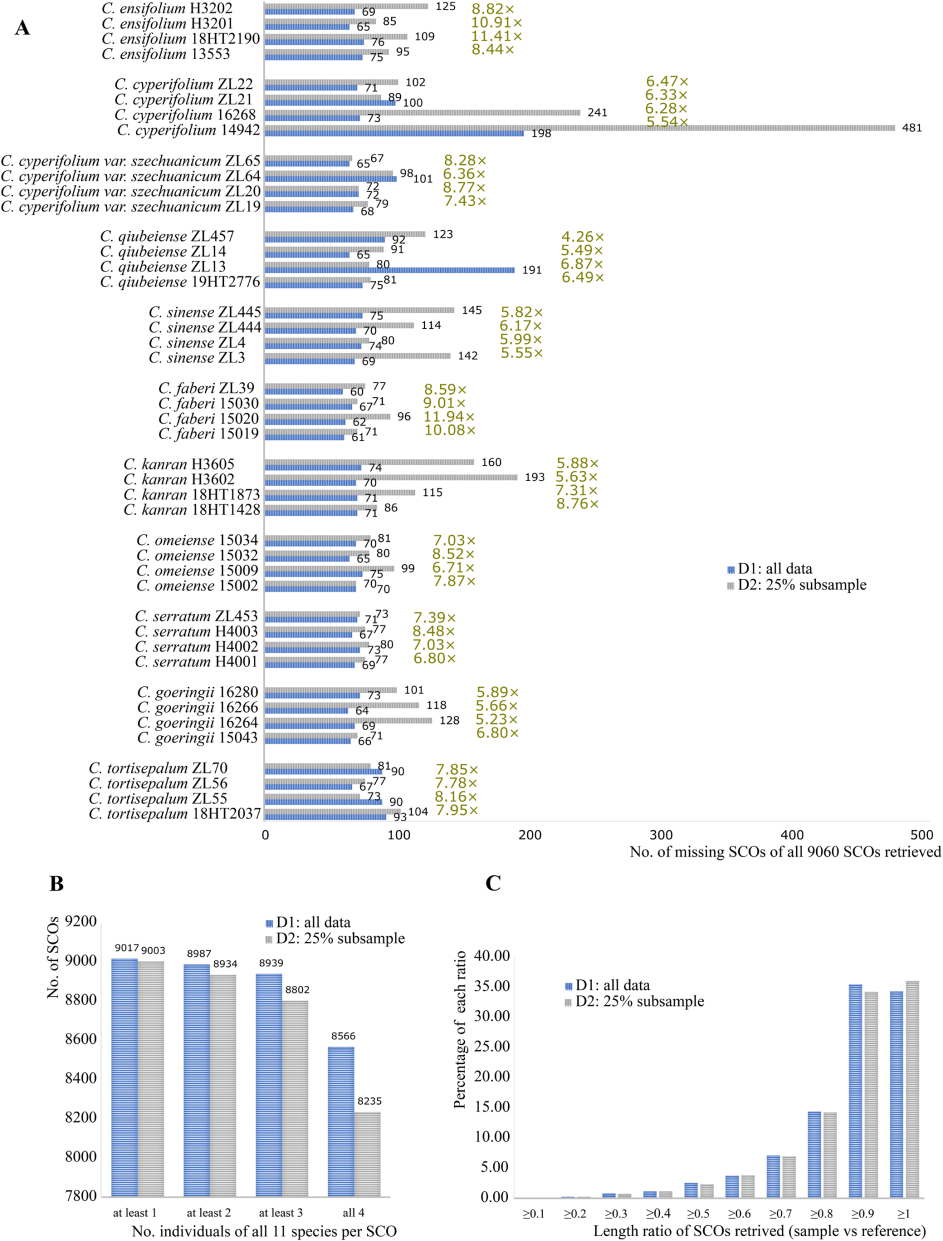
Performance metrics of AliBaSeq. (A) The count of missing SCOs from the total of 9060 SCOs extracted for each individual in data sets D1 and D2. (B) The number of SCOs extracted across all species for each SCO. (C) The frequency distribution of the ratio of the mean length of retrieved SCOs to the mean length of corresponding reference SCOs.

The average assembly sizes for all 44 individuals, based on approximately 100 Gb of data (hereafter referred to as D1) and 25% subsampled data (hereafter referred to as D2), were 7.18 Gb and 3.75 Gb, respectively. Notably, the smallest assembly sizes observed for *C. cyperifolium* 14942 (1.56 Gb in D1 and 0.4 Gb in D2, respectively) likely resulted from an extremely high duplication rate during genomic sequencing, suggesting that the actual depth of voucher 14942 might be significantly lower than 22.14× (Table S1). ALiBaSeq successfully retrieved 9060 putative SCOs from each data set, with only 34 reference SCOs yielding no hits in both D1 and D2, and only 2 putative SCOs differing between the data sets. For each species, 98.95% and 98.06% of the total 99,660 SCOs (9060 multiplied by 11) were obtained across all four individuals from data sets D1 and D2, respectively. The total number of individuals per gene displayed a slight correlation with the length of reference SCOs (Tables S2 and S3). On average, 99.5% and 99.2% of SCOs were successfully retrieved from each individual in data sets D1 and D2, respectively, with the lowest efficiency observed in *C. cyperifolium* 14942 (Figure 2A). From the perspective of SCO analysis, 9017 and 9003 out of 9060 putative SCOs were acquired from at least one individual of each species in data sets D1 and D2, respectively. Additionally, 8566 and 8235 out of 9060 putative SCOs were retrieved from all four individuals of each species in data sets D1 and D2, respectively (Figure 2B). The ratios of the mean lengths of retrieved putative SCOs to the mean lengths of corresponding reference SCOs were generally greater than 0.9, with accumulative frequencies of 69.8% and 70.3% in D1 and D2, respectively (Figure 2C, Table S4). Overall, ALiBaSeq demonstrated excellent performance in both the efficiency of recovery and the representativeness of the recovered SCOs.

### 3.3 Nonhomologous Alignment Issues Were Alleviated Using Spruceup

**FIGURE 3:**
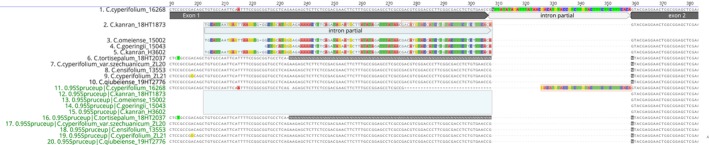
Addressing nonhomologous alignment caused by introns. Highlights how nonhomologous alignment, for example, in gene9094.1473, can be mitigated by using Spruceup. Blue shadows indicate misaligned intron residual sequences intertwined with exon sequences. Red‐bordered rectangles pinpoint nucleotides requiring realignment after Spruceup filtering.

The accuracy of phylogenetic reconstruction hinges on the correct identification of homologous sites via sequence alignment. Credible phylogenetic trees are derived only from homologous alignments. Residual intron sequences incorrectly aligned with neighboring exon sequences can disrupt homology, resulting in nonhomologous alignments. This issue can be mitigated by adjusting the scoring matrix in TBLASTN from the default BLOSUM62 to PAM30, which is effective in excluding intron sequences (Figure S3). After alignment with MAFFT, the aligned matrix can be further refined using Spruceup, which not only reduces the Shannon entropies of the alignment but also increases the average pairwise sequence identity (Figure 3). However, even with these enhancements, the resultant alignments from Spruceup may require realignment to achieve fully refined alignments (Figure S3).

### 3.4 SCOs Outperform Plastomes and rDNA

**FIGURE 4:**
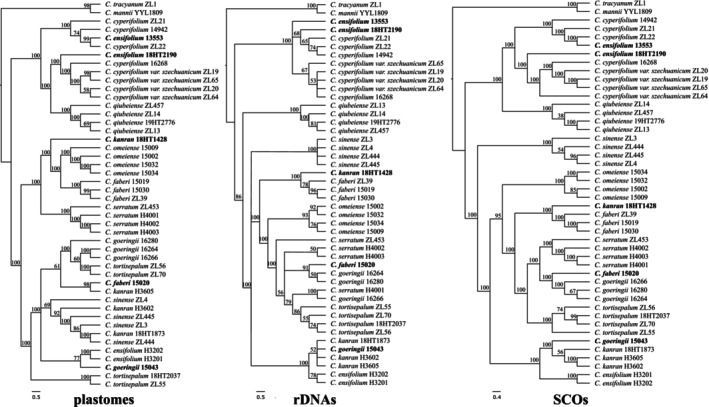
Cladogram for species discrimination within *Jensoa* constructed from various data sets. Specimens that are potentially misidentified are highlighted in bold. The percentages above each branch represent SH‐like (Shimodaira‐Hasegawa) local support values for plastome and rDNA trees, and LPP (local posterior probability) for SCO trees (reconstructed using 6083 SCOs with APSI ≥ 85%).

Our prior work demonstrated that the identification rate of *C*. subg. *Jensoa* was the lowest among the genus *Cymbidium* using plastomes as a barcode (Zhang, Huang, et al. 2023). After curation of the plastomes from 44 individuals across 11 species in this study, *Cymbidium cyperifolium* var. *szechuanicum* and *Cymbidium serratum* were successfully identified. Although rDNA clusters managed to identify *C. tortisepalum* and *C. sinense* , which plastomes could not, they failed to identify *C. cyperifolium* var. *szechuanicum* and *C. serratum*. SCOs (extracted from data set D1 and two outgroup) surpassed both rDNA clusters and plastomes in performance; however, *C. ensifolium*, *C. kanran*, *C. faberi*, and *C. goringii* did not form monophyletic clades (Figure 4). Species trees reconstructed from SCOs extracted from data set D1 (full data) and D2 (25% subsample) exhibited identical topology and branch support values (Figure S1). This finding indicates that deep genome skimming (DGS) with as low as 4–5× coverage is sufficient for ALiBaSeq to recover a substantial number of SCOs, enabling the construction of robust species trees. Notably, ALiBaSeq exhibited superior performance compared to HybPiper, even with half the sequencing depth (Liu, Ma, Ren, et al. 2021). It is important to highlight that the four species where SCOs identification failed also showed anomalies in trees reconstructed by plastomes and rDNA clusters. The discrepancies primarily originate from taxonomically misclassified vouchers exhibiting morphological congruence (molecular materials in bags), specifically vouchers 18HT1428, 15020, and 15034 (Figure 3, Figure S1), rather than inherent limitations in the molecular approaches employed. Including additional vouchers is essential to resolve these discrepancies.

### 3.5 Enhancing Validation of SCOs Through Additional Sampling

**FIGURE 5:**
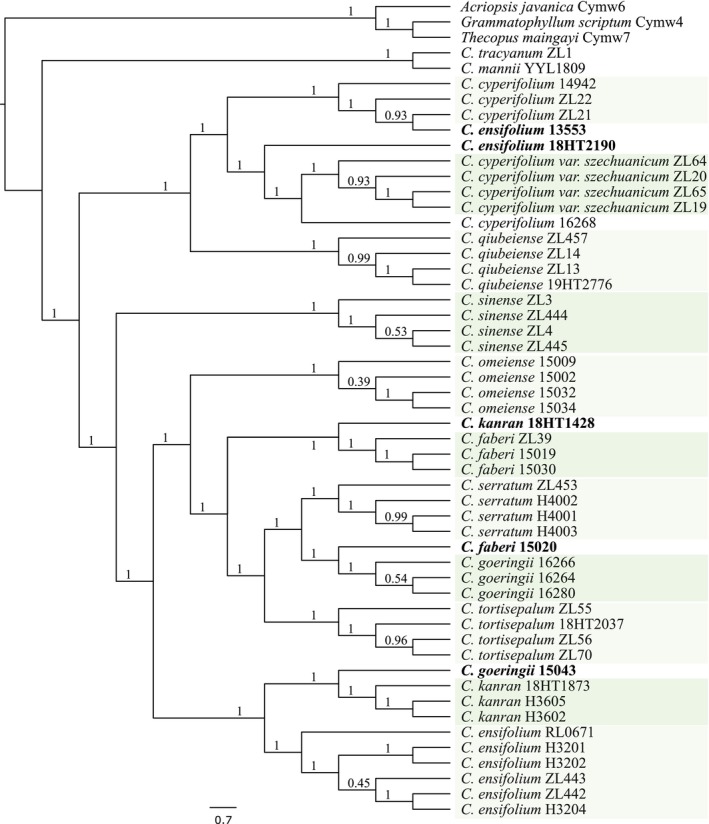
Species tree construction from SCOs. Species tree of 11 *Cymbidium* species reconstructed using 5732 SCOs with APSI ≥ 85%. Numerical values above each branch, expressed as decimals, represent the LPP (local posterior probability). Specimens potentially misidentified are highlighted in bold.

Upon incorporating four additional vouchers of *C. ensifolium* and three vouchers representing distantly related outgroups into data set D2, the effectiveness of SCOs was confirmed. Notably, two vouchers of *C. ensifolium*, 13553 and 18HT2190, were initially misidentified, as they were genetically distant from the other six *C. ensifolium* accessions, suggesting they might actually be *C. cyperifolium* or *C. cyperifolium* var. *szechuanicum*. Four individuals of *C. cyperifolium* var. *szechuanicum* consistently formed a monophyletic clade, as opposed to *C. cyperifolium* (Figure 5). This study replicated the genomic data of vouchers by conducting all the molecular experiments anew, including those used in our previous research. Three vouchers, namely 18HT1428, 15020, and 15034, which exhibited confusion in identification, might have been incorrectly identified or mislabeled prior to the molecular analyses. Additionally, in a previous study, vouchers 18HT1428 and 15020 were associated with *C. faberi* and *C. kanran*, respectively (Zhang, Huang, et al. 2023). Further inclusion of vouchers is necessary to substantiate these findings, emphasizing the need for meticulous curation of the voucher collection database by experts. Removal of these five problematic vouchers resulted in all conspecific samples forming reciprocal monophyletic groups, with the exception of *C. cyperifolium* (voucher 16268) (Figure S2). It is crucial to recognize that single copy orthologs (SCOs) have effectively distinguished every species within *C*. subg. *Jensoa*, demonstrating their potential as a powerful barcode for species identification at lower taxonomic levels. This is particularly valuable in situations characterized by recent divergence or ancient rapid radiation, which may limit sequence variation.

## 4 Discussion

### 4.1 Effective at Low Sequencing Depths Below 10×

The number of SCOs recovered by HybPiper decreases significantly when genomic sequencing depth falls below 10×, particularly with an average nucleotide coverage cutoff value of 5 (Liu, Ma, Ren, et al. 2021). This reduction is likely due to the reliance on the integrated assembling software SPAdes, which is primarily designed for assembling small genomes, such as those of microorganisms. By default, HybPiper conducts per‐sample/gene assemblies using SPAdes with the parameter “–cov‐cutoff 8,” aiming to produce shorter contigs while maintaining high base‐level accuracy (Johnson et al. 2016). However, reducing the “–cov‐cutoff” value to 5 does not improve outcomes at coverages below 10× (Liu, Ma, Ren, et al. 2021). Unlike HybPiper, ALiBaSeq does not assemble reads mapped to reference SCOs but delegates the task of whole‐genome assembly to specialized software designed to handle complex genomes with large sizes and high repetitive element content. The actual sequencing depth of a 25% subsampled *C. cyperifolium* 14942 may be less than 3× due to its exceptionally high PCR duplication rate of 59.5% (Table S1). Despite this, only 481 of the 9060 SCOs failed to be recovered (Figure 2A). Utilizing lower sequencing depths not only reduces financial costs but also eases computational demands.

### 4.2 Efficient and Effective Pipeline for Convincing SCO Matrices

To construct convincing SCOs matrices for species tree reconstruction, we explored and compared a variety of software solutions. Unlike GenomeScope2 (Ranallo‐Benavidez et al. 2020) or FindGSE (Sun et al. 2017), which both require a genome sequencing depth of over 30×, RESPECT requires only a minimal sequencing depth of 0.5×–4× to estimate sample's genome size (Sarmashghi et al. 2021), making it more applicable to genome skimming data. This also allows researchers to downsample genomic sequencing data incrementally to obtain stable values calculated by RESPECT. Although RESPECT estimates were, on average, approximately 1.19 times larger than flow cytometry measurements, similar estimates could be obtained by eliminating this bias. We also recommend MegaHit for its consistent performance and reduced memory usage, after evaluating it alongside other resource‐efficient whole‐genome assembly software such as SOAPdenovo2 (Luo et al. 2012), Minia3 (https://github.com/GATB/minia), and SH assembly (Shi and Yip 2020). Unlike HybPiper, which cannot directly extract SCOs from available genome assemblies, ALiBaSeq is capable of retrieving SCOs from existing genome assemblies, regardless of whether annotations are available. However, assembling a whole genome requires substantial computing resources. We were unable to successfully run HybPiper v1.3 on the *Jensoa* data set, but we did test it on *Arabidopsis* (unpublished data), where ALiBaSeq significantly outperformed HybPiper at sequencing depths below 10×, corroborating previous findings (Liu, Ma, Ren, et al. 2021). Nonetheless, with the recent release of HybPiper v2, its performance needs re‐evaluation. Another software worth exploring is Easy353 (Xie, Guo, et al. 2024; Zhang et al. 2022), particularly since aligning nucleotides of orthologous introns can be challenging, especially when samples are relatively distantly related (Creer 2007; Sverdlov et al. 2005). Our study also reveals that protein‐coding regions of SCOs suffice for constructing high‐resolution species trees, and that introns of SCOs are not necessary. In terms of alignment refinement, Spruceup surpasses other popular software, such as Gblocks (Castresana 2000), trimAl (Capella‐Gutiérrez et al. 2009), and MACSE (Ranwez et al. 2018), providing more effective results.

### 4.3 Maintaining Rigorous Percent Identity in SCO Alignments

A commonly accepted benchmark asserts that two sequences are considered homologous if they share more than 30% identical nucleotides across their full lengths (Pearson 2013). For phylogenetic analyses involving low‐copy nuclear genes, a sequence identity of 60% is advised, particularly for encoded proteins comprising at least 300 amino acids (Zhang et al. 2012). For accurate species tree reconstruction via ASTRAL, it is optimal to retain as many SCOs as possible (Warnow 2015). In our study, stringent criteria for SCO alignments were applied. We observed that approximately half of all retrieved SCOs satisfied the criterion with an average pairwise sequence identity (APSI) ≥ 80%. Further testing involved using all SCOs without percent identity filtering, as well as SCOs with APSI exceeding 90% and 95%. The resulting topologies of the species trees were almost identical, although the local posterior probability (LPP) support values showed a slight decrease.

### 4.4 Perspectives on the Use of SCOs

Organellar genomes are primarily inherited uniparentally, while rDNA genes, having a high copy number, are prone to incomplete homogenization. Conversely, low‐copy orthologous nuclear genes provide a biparental record essential for revealing evolutionary history. For an accurate resolution of relationships among closely related species, utilizing more nuclear genes—encompassing both genes with slow and rapid evolutionary rates—is recommended (Li et al. 2017; Sang 2002; Zhang et al. 2012). Unlike the findings of Dowton et al. (2014), our empirical data compellingly illustrate that multilocus coalescent methods surpass DNA barcoding in effectiveness (Collins and Cruickshank 2014).

Considering scenarios with limited closely related genomes, could predetermined orthologous gene sets be utilized? OrthoDB v5 catalog groups of orthologous genes hierarchically, from broader lineages to more specific delineations (Kriventseva et al. 2019). We evaluated 1614 SCOs from the embryophyta_odb10 data set (derived from 50 land plant genomes), following the same workflow as the 9094 baits. The resulting species tree constructed from 709 SCOs of the 1614 set closely resembled the tree derived from 5648 SCOs of the 9094 set, except for the sample *C. ensifolium* RL0761 (Figure S2). OrthoDB proves a reliable source for SCOs when close genomic annotations are unavailable. Other SCO sets, such as the Angiosperms353 gene set (Johnson et al. 2018), or the strictly/mostly single‐copy OGs utilized by MarkerMiner (Chamala et al. 2015; De Smet et al. 2013), could also be considered.

Compared to targeted sequencing, deep genome sequencing offers a promising avenue for generating extensive data sets of SCOs in silico, bypassing the need for labor‐intensive bait synthesis and complex target enrichment. In this study, both the predefined embryophyte_odb10 and the 9094 SCOs inferred from three *Cymbidium* genomes demonstrated sufficient resolution at lower taxonomic levels (Figure S2). This raises the question: Could SCOs serve as new universal markers across the entire plant kingdom, similar to standard barcodes (Li, Yang, et al. 2015)? OrthoDB‐like SCOs, termed universal single‐copy orthologs (USCOs), which can be inferred from thousands of available genomes across varied plant levels, might be a significant resource for screening user‐friendly barcodes applicable to both high‐ and low‐rank taxonomic hierarchies (Eberle et al. 2020).

To fully harness and validate the potential of SCOs as the next generation of DNA barcodes, it is crucial to include more recently diverged species and additional vouchers per species. Moreover, numerous aspects of phylogenetics, molecular evolution, and population genetics stand to gain substantially from resources of putative SCOs. The most significant costs associated with DNA barcoding include sample collection, prelaboratory processing, DNA isolation, databasing, and curation of vouchers in collections (Matz and Nielsen 2005). Sequencing library preparation and next‐generation sequencing (NGS) do not require additional investments compared to DNA barcode versions 1.0 and 2.0, especially as the costs of parallel sequencing continue to decline (Mallo and Posada 2016). Given the rapid improvements in bioinformatic tools and computational resources, multilocus species trees constructed from SCOs are poised to become prevalent in applications such as specimen identification, species delimitation, hybrid speciation, infra‐species structuring, and others.

## Author Contributions

**Zheng‐Shan He:** data curation (lead), formal analysis (lead), methodology (lead), software (lead), visualization (lead), writing – original draft (lead), writing – review and editing (equal). **Ji‐Xiong Yang:** resources (equal), writing – review and editing (supporting). **Jia‐Lin Huang:** resources (lead), writing – review and editing (supporting). **De‐Zhu Li:** project administration (lead), supervision (equal), writing – review and editing (supporting). **Jun‐Bo Yang:** conceptualization (lead), funding acquisition (lead), project administration (lead), supervision (lead), writing – review and editing (lead).

## Conflicts of Interest

The authors declare no conflicts of interest.

## Supporting information


Figure S1.

Table S1.

## Data Availability

All raw data generated in the current study are available at the National Genomic Data Center of the China National Center for Bioinformation under project number PRJCA031100. The assembled plastome, rDNA, recovered putative SCOs alignments, and species trees have been deposited in Figshare (https://doi.org/10.6084/m9.figshare.27840468).
