# Supplementary material for: Specimen Identification Through Multilocus Species Tree Constructed From Single‐Copy Orthologs (SCOs): A Case Study in Cymbidium Subgenus Jensoa
Source: Ecol Evol. 2025 Apr 24;15(4):e71323. doi: 10.1002/ece3.71323 (PMC12019701; doi:10.1002/ece3.71323)
Supplement: Supplementary file 1 — Figure S1. [file ECE3-15-e71323-s002.pdf]

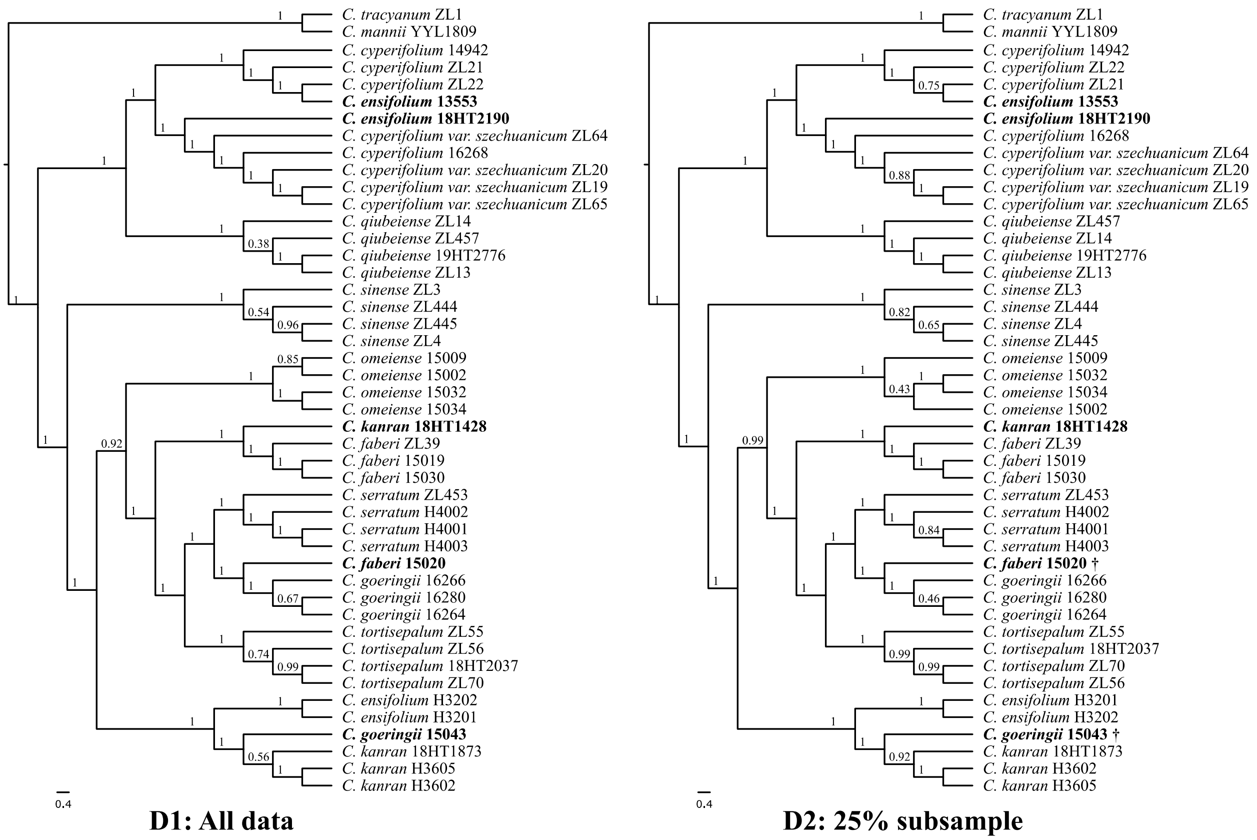

Supplementary FIGURE 1. Performance Comparison of SCOs from Two Data Sets. Displays a comparative analysis of SCOs recovered from dataset D1 (full data set) and D2 (25% subsample). Trees were reconstructed using 6083 SCOs from D1 and 5991 SCOs from D2, each with an Average Pairwise Sequence Identity (APSI) of ≥ 85%. Numerical values above each branch, expressed as decimals, denote the Local Posterior Probability (LPP). Vouchers that are potentially misidentified are highlighted in bold.

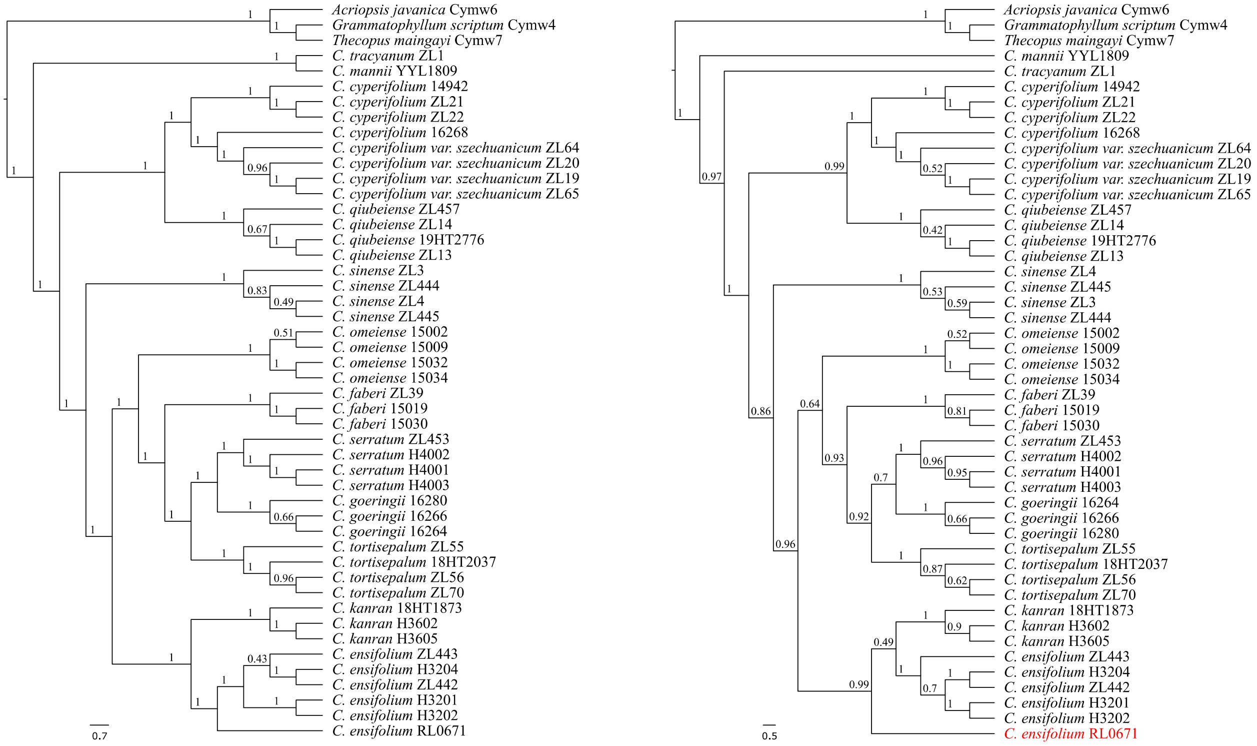

A: SCOs from *Cymbidium* **B: SCOs from embryophyta_odb10**

Supplementary FIGURE 2. Species Tree Reconstruction with Filtered SCOs. This figure illustrates the species tree reconstructed using 5648 SCOs (selected from a total of 9094 SCOs, with an Average Pairwise Sequence Identity (APSI) of ≥ 85%) from Cymbidium, alongside 709 SCOs (selected from a total of 1614 SCOs, with APSI ≥ 85%) from embryophyta_odb10. The reconstruction follows the removal of five vouchers identified as mislabeled. Numerical values above each branch point, expressed as decimals, represent the Local Posterior Probability (LPP). Notably, one voucher, highlighted in red, is not clustered with the other vouchers from *C. ensifolium*, suggesting a potential outlier or misidentification.

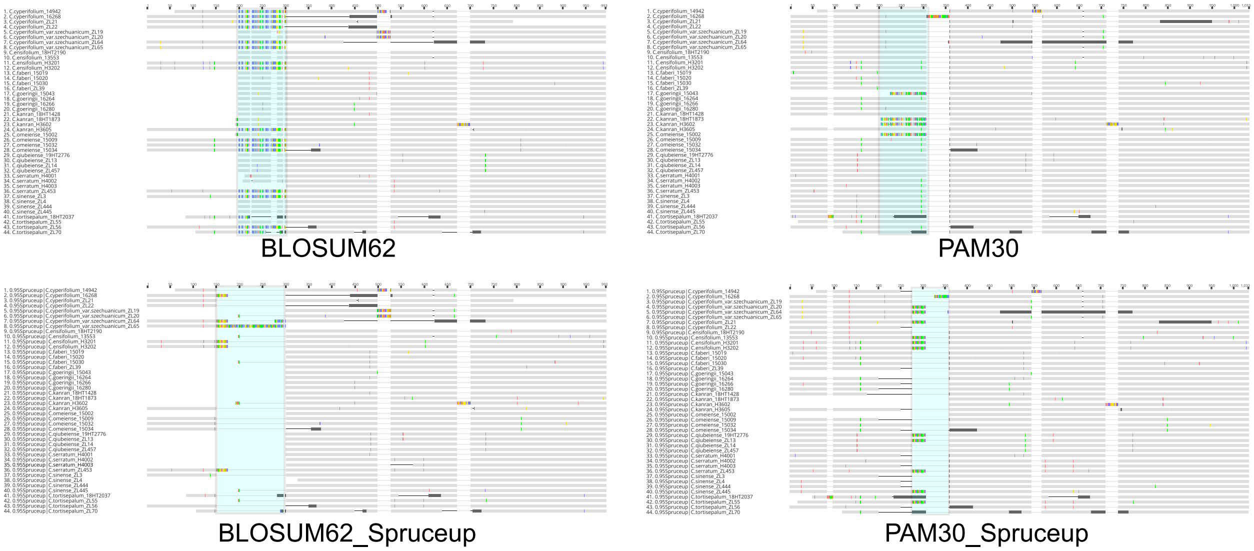

Supplementary FIGURE 3. Mitigation of Nonhomologous Alignment Due to Introns. This figure demonstrates how the issue of nonhomologous alignment, exemplified by gene9094.1473, can be alleviated by altering the scoring matrix and applying filtering with Spruceup. The blue shadows highlight areas where misaligned intron residual sequences are intermingled with exon sequences, illustrating the effective segregation achieved through these adjustments.
